# The effect of current flow direction on motor hot spot allocation by transcranial magnetic stimulation

**DOI:** 10.14814/phy2.12666

**Published:** 2016-01-05

**Authors:** Caspar Stephani, Walter Paulus, Martin Sommer

**Affiliations:** ^1^ Department of Clinical NeurophysiologyUniversity of GöttingenGöttingenGermany

**Keywords:** Motor mapping, pulse configuration, TMS

## Abstract

The objective of this study was to investigate the significance of pulse configurations and current direction for corticospinal activation using transcranial magnetic stimulation (TMS). In 11 healthy subjects (8 female), a motor map for the motor evoked potentials (MEPs) recorded from the first dorsal interosseus (FDI), abductor digiti minimi (ADM), extensor carpi radialis, and biceps brachii (BB) muscles of the dominant side was established. Starting from a manually determined hot spot of the FDI representation, we measured MEPs at equal oriented points on an hexagonal grid, with 7 MEPs recorded at each point, using the following pulse configurations: posteriorly directed monophasic (Mo‐P), anteriorly directed monophasic (Mo‐A), biphasic with the more relevant second cycle oriented posteriorly (Bi‐P) as well as a reversed biphasic condition (Bi‐A). For each pulse configuration, a hot spot was determined and a center of gravity (CoG) was calculated. We found that the factor current direction had an effect on location of the CoG‐adjusted hot spot in the cranio‐caudal axis but not in the latero‐medial direction with anteriorly directed pulses locating the CoG more anteriorly and vice versa. In addition, the CoG for the FDI was more laterally than the cortical representations for the abductor digiti minimi (ADM) and extensor carpi radialis (ECR) which were registered as well. The results indicate that direction of the current pulse should be taken into account for determination of the motor representation of a muscle by TMS.

## Introduction

Three main types of pulse configuration – monophasic, half‐sine and biphasic pulses – are used in studies applying TMS (Sommer et al. 2006). Triggering pulse configurations and current direction of single transcranial magnetic pulses influence resting and active motor threshold, latency of motor evoked potentials (MEP), cortical silent period and aftereffects of repetitive TMS (Kammer et al. 2001; Orth and Rothwell 2004; Sommer et al. 2006, 2013). Regarding pulse configuration, resting, and active motor threshold are both lower upon biphasic pulses compared to monophasic pulses, with half‐sine pulses being of intermediate effectiveness (Kammer et al. 2001; Sommer et al. 2006). With respect to pulse direction, the lower thresholds for eliciting MEPs are associated with anteriorly directed monophasic pulses rather than posteriorly directed ones (Sommer et al. 2006). The same effect is observed for biphasic pulses referring to the second cycle as the physiologically relevant one. Thus biphasic pulses, being directed first posteriorly and anteriorly in the second cycle demand the overall lowest current intensity to induce an MEP. On the other hand, it has been argued that monophasic currents lead to more focal corticospinal activation than biphasic pulses (Brasil‐Neto et al. 1992). Moreover, the choice of pulse configuration and pulse direction influences results of repetitive TMS (rTMS). Compared to biphasic stimulation, low‐frequency rTMS with monophasic pulses was found to induce a more profound inhibitory effect on the visual cortex as measured by the static contrast sensitivity (Antal et al. 2002), and throughout on the motor cortex as measured by the amplitudes of the MEPs (Sommer et al. 2002). However, waveform and current direction were not found to influence the effectiveness of theta‐burst‐stimulation protocols, a subform of rTMS (Zafar et al. 2008). Functionally, anteriorly directed currents stimulate the cortex from layer I to layer VI while posteriorly directed currents stimulate from layer VI to I (Jefferys 1981).

TMS is used to map the primary motor cortex and has repeatedly demonstrated results that are in accordance with data from direct cortical stimulation (Penfield and Boldrey 1937) and neuroimaging techniques like fMRI (Hlustík et al. 2001) and PET (Grafton et al. 1991), confirming varying degrees of somatotopic patterns as well as functional segregation in the motor cortex (Wassermann et al. 1992; Z'Graggen et al. 2009). One option with respect to such non‐invasive motor‐mapping is the calculation of a center of gravity (CoG), which allows the most excitable area of a given muscle representation to be delineated. Calculating the center of gravity takes into account MEPs that have been evoked from a number of adjacent points and weighs them based on their distance within a reference matrix (Wassermann et al. 1992; Littmann et al. 2013). In such studies, the motor mapping was generally performed by using a fixed single pulse configuration depending, for example, on the available stimulator. Given the aforementioned differences in electrophysiological outcome measures depending on pulse configuration and direction, we hypothesized that the centers of gravity of the first dorsal interosseous muscle (FDI) differ depending on pulse configuration and current direction – namely monophasic (Mo) and biphasic (Bi) pulses with posteriorly (P) or anteriorly (A) oriented main currents. To assess spread of activation to adjacent muscle representations, the EMG of the abductor digiti minimi (ADM), the extensor carpi radialis (ECR) as well as the biceps brachii (BB) of the same extremity were also recorded.

The direction of the pulse as indicated in this article per convention always refers to the current flow direction in the brain. Thus, the term Mo‐P indicates a monophasic pulse directed from anterior to posterior within the brain and the term Mo‐A the reverse, while Bi‐P and Bi‐A are used similarly for biphasic pulses acknowledging the second and physiologically more relevant second cycle.

## Materials and methods

### Subjects

In this study, 11 right‐handed subjects (8 female) aged 21–38 years (mean age 27.45 ± 6.19 years) were tested. Persons aged 18 or older were eligible. Exclusion criteria were previous or ongoing disease of the CNS, electrical implants or metallic material inside the head or neck, cardiac pacemaker, pregnant, or lactating women. The study was approved by the ethics committee of the University of Göttingen, according to the declaration of Helsinki, including the amendment from Edinburgh (2000). All subjects gave their informed consent prior to participation in the study.

### TMS

**Figure 1:**
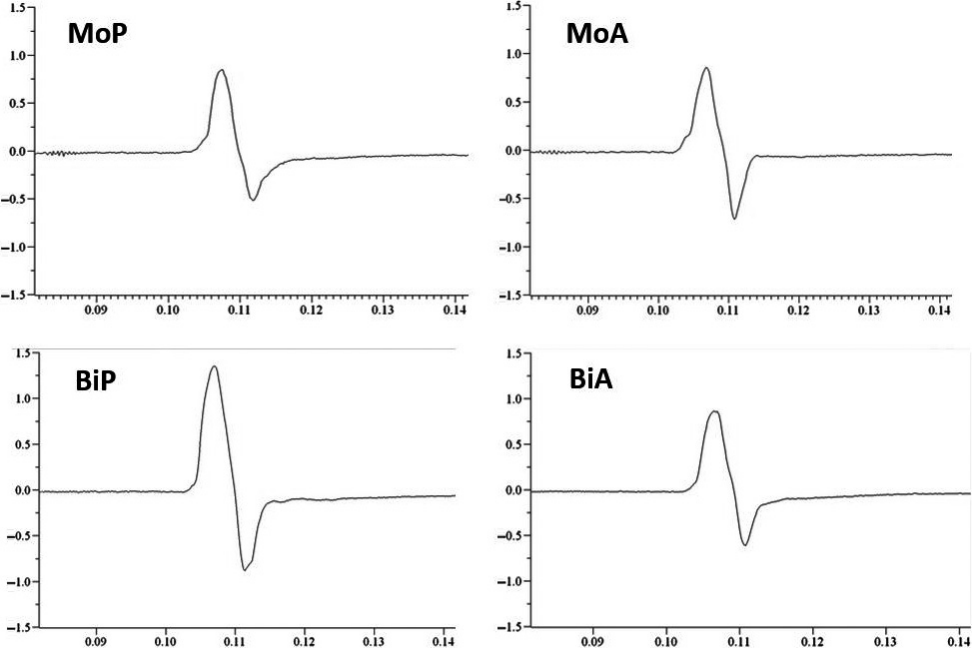
Exemplary motor evoked potentials of the first dorsal interosseous muscle after stimulation with a monophasic posteriorly (MoP), a monophasic anteriorly (MoA), a biphasic posteriorly (BiP) and a biphasic anteriorly directed pulse. The *Y*‐axis is in Volt and the *X*‐axis displays seconds.

Motor evoked potentials (MEP) were recorded via a surface electromyogram (EMG) from the right FDI, the ADM, the ECR, and the BB muscles (all right‐handed). In each participant, one custom‐built silver‐chloride electrode was placed over the belly of the muscle itself and the other electrode as a reference about 3–5 cm away over a tendon or joint (electrode impedance = 100 Ω – 10 kΩ, low‐pass filter = 2.5 kHz, sampling‐frequency = 5 kHz). For amplification we used an isolated patient amplifier system (Digitimer, Ltd., Model D360, Garden City, UK) and an analogue‐digital‐converter (CED 3001, MICRA 1401 mk II; Cambridge Electronic Design Limited, Cambridge, UK). For EMG recording we used the software “Signal” (CED 1401, Cambridge Electronic Design, Cambridge, UK). Cortical stimulation was performed with a slightly angulated TMS figure‐of‐eight coil with an outer diameter of each wing of 7 cm (MC B70, Dantec S.A., Skovlunde, Denmark), which was connected to a MagPro X 100 Mag Option stimulator^®^ (Medtronic Inc., Minneapolis, MN). The options for setting the triggering pulses allowed choosing the pulse direction (anterior to posterior vs. posterior to anterior) as well as the pulse configuration (monophasic vs. biphasic) with the duration of monophasic pulses being approximately 40 *μ*s and that of biphasic pulses 32 *μ*s (Sommer et al. 2006). Exemplary MEPs induced by the different pulse conditions are shown in Figure 1. The coil was placed tangentially to the skull and at a 45° angle to the sagittal midline. The basic measurements included the resting motor threshold (RMT) and the pulse intensity needed to achieve an MEP amplitude of 1‐mV on average. The RMT was determined as the minimal TMS intensity that evoked EMG amplitudes of at least 50 *μ*V in at least 5 of 10 TMS pulses from peak‐to‐peak. The test pulse intensity was determined as the mean of 20 consecutive pulses in which individual intensity elicited MEPs with amplitude of approximately 1 mV. Values of more than 5 mV amplitude were excluded from the calculation. The stimulator was triggered by protocols for this study written in the software Signal 2.16 (Cambridge Electronic Design).

### Experimental design

**Figure 2:**
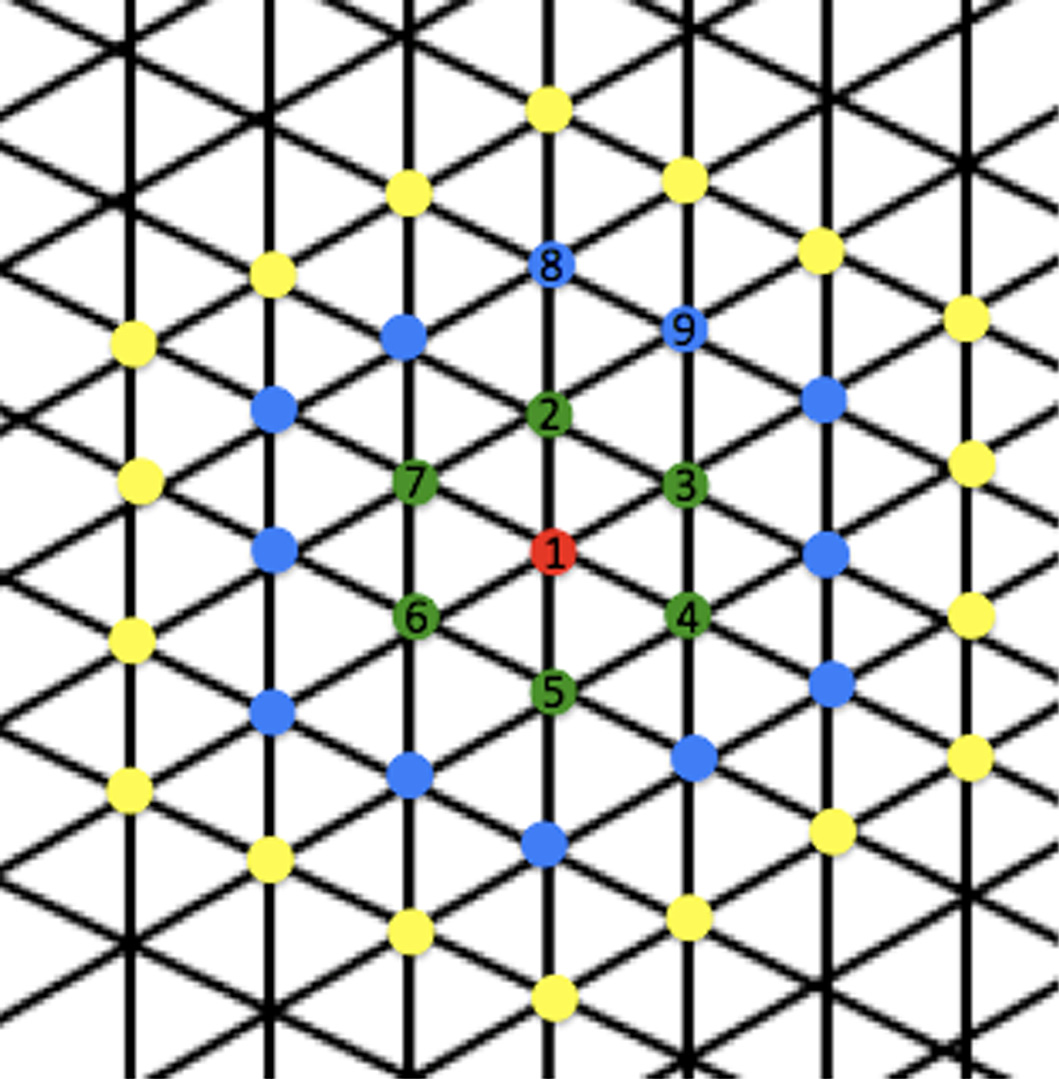
Matrix used for determination of the hot spot. From the center (red dot), found by manual search as the optimal representation of the FDI, the surrounding dots were measured successively by first moving to the green point in front of the red point with respect to the median‐sagittal line and continuing clockwise as indicated by the numbers. The first 37 stimulation points around a pre‐determined “hot spot” are depicted in color and were stimulated in each participant and for each condition. In most cases considerably more points were stimulated. The distance between each neighboring point is 5.77 mm.

As primary target muscle of this study we chose the FDI. Hence, we always searched for the primary representation of this muscle while the EMGs of the other muscles (ADM, ECR, BB) were also recorded. All further single pulse TMS measurements per stimulation mode referred to the baseline value of the TMS intensity that elicited an amplitude of about 1 mV in the FDI. This procedure was repeated for each stimulation mode. For a structured motor mapping, we used a physical hexagonal grid, drawn on a self‐fixing paper that was pasted onto a small tightly fixed bath cup. The high‐density grid of equidistant points being located 5.77 mm from each other was used as matrix based on isosceles triangles with a side length of 5.77 mm and a height of 5 mm (calculated as 2*h/√3). This distance was chosen to achieve a maximal resolution for each muscle being close to the optimal and lowest possible distance of 5 mm (Brasil‐Neto et al. 1992). Approximately in the center of the hexagonal grid, the assumed optimal or primary representation of the FDI had been determined manually based on a systematic search with the TMS‐coil in all directions. This point then was marked with a colored pencil. From there on, we measured 7 MEPs at 0.25 Hz at consecutive points clockwise and oriented radially from the hot spot according to Figure 2, with equidistance between all neighboring points and always starting from posterior to anterior in the midline up until at least stimulation point 37 while the TMS pulse rate of 0.25 Hz ran continuously.

The motor mapping was stopped when the examiner judged that there were no discernible MEPs evoked in the EMG at a number of consecutive points. To account for interindividual differences in the motor map the order of stimulation points after point 37 was variable and depended on where and to which extent MEPs could be evoked from this point onwards. The coil‐orientation of 45° aside from the sagittal plane on the dominant hemisphere and close to the optimal coil presentation of 50° for stimulating the motor cortex (Mills et al. 1992) was manually maintained throughout the whole mapping procedure. This procedure was performed with four different stimulus configurations: Mo‐P, Mo‐A, Bi‐A and Bi‐P (Sommer et al. 2006). The order of these configurations was balanced between participants to eliminate possible sequence effects. Stimulation was performed within a single session for each pulse configuration. There were breaks of 5–15 min between the stimulation protocols, which lasted 2.5–4 h in total. The subjects were comfortably seated in a dentist chair with an individually adjustable headrest to minimize lateral head movements.

To create a common coordinate system we referred the measurements to the hot spot of the Mo‐P condition representing our arbitrary reference condition. Distances between the manually evaluated Mo‐P hot spot and the Mo‐A‐, Bi‐A‐, and Bi‐P hot spots were determined by the differences in their Cartesian coordinates in the x‐ and y‐direction, respectively. Since the hot spots of all four conditions had been marked in the same hexagonal grid, their inter‐hot spot distance could be measured easily thus giving every hot spot a coordinate distance with respect to that of the Mo‐P condition. The calculated coordinates for the CoG for each condition and as described below were then added to the manually determined hot spot coordinates for each participant, resulting in a final hot spot. Mean and standard deviations for the whole group were then calculated. Values are given in cm. In determining the final hot spot using the CoG correction, only nine of the total of 11 subjects were considered since the single hot spots of two subjects were measured in separate grids which affected the accuracy of the measurements of the relative distances between the hot spots for the different stimulation modes. In addition, due to the long duration of the procedure we had to leave out one condition with particularly extensive measurements in three participants. Therefore, we have a complete set of 11 stimulations for the Mo‐P configuration, whereas there are 10 complete measurements for the other conditions of Mo‐A, Bi‐A, and Bi‐P. Therefore, we had eight participants completing all test conditions.

### Statistics

#### Basic measures of excitability

The statistical analyses comparing the baseline parameters RMT and 1 mV thresholds were each performed as a repeated measures analysis of variance (ANOVA). The value of the MEP threshold was determined by the percentage of the maximum stimulator output and represented the dependent variable. Pulse configuration (monophasic vs. biphasic) and pulse direction (anterior–posterior vs. posterior–anterior) were the independent factors. Paired two‐sample *t*‐tests corrected for multiple testing were performed for post hoc testing. Box‐plots were chosen for presentation of descriptive statistics.

Since the absolute number of stimulated matrix points differed for each subject and each pulse condition we decided to use two sets of data for the calculation of the size and location of the motor representation as indicated. One calculation includes all matrix points stimulated (ranging from 41 to 166 in total) and another calculation including a subset of the first 37 matrix points only. These were identically represented between individuals.

#### Size of motor representation

In another univariate repeated measures ANOVA we compared the total number of stimulated points (dependent variable) between pulse configurations with the independent factors pulse configuration (monophasic vs. biphasic) and pulse direction (anterior–posterior vs. posterior–anterior). In addition, the sum of the mean of the MEP‐amplitudes (dependent variable) of the first 37 and all matrix points, respectively – and as indicated above – were tested in a repeated measures ANOVA with three factors: pulse configuration (monophasic vs. biphasic), pulse direction (anterior–posterior vs. posterior–anterior) and muscle (FDI, ADM, ECR, BB) being the independent variables. Again, paired two‐sample t‐tests corrected for multiple testing were performed for post hoc testing.

#### Location of the motor representation

##### “Primary” hot spot

The location of the hot spot of the Mo‐P‐directed pulses represented our arbitrary reference point (0;0) in a relative coordinate system. The distances to the hot spots of the other three pulse modalities were measured. Hence, the mean hot spots for the Mo‐A, Bi‐A and Bi‐P conditions were calculated as averages of the distances to the hot spot of the Mo‐P condition based on their matrix coordinates. Since we used a single grid for all 4 stimulation modes (from the third participant onwards), we were able to include 9 participants' data into this analysis. Repeated measures ANOVAs with the x‐ and y‐coordinates as the dependent variables and pulse configuration (monophasic vs. biphasic) and pulse direction (anterior–posterior vs. posterior–anterior) as independent factors were performed to test for systematic differences in the manually determined hot spots.

##### CoG

For calculation of the CoG we multiplied the amplitude of the MEP at a given point with its x‐ and y‐coordinate of the matrix used during stimulation. Each result was divided by the total sum of MEP amplitudes of a given matrix. The final coordinate was then determined by the sum total of all calculated points, indicating the amount of deviation from the starting point (Wassermann et al. 1992). This calculation was performed twice for all 11 participants, first with the central 37 matrix points that were identically placed in all the subjects and second with all available stimulation points representing a range from 41 to 166 matrix points in total.

##### “Final” hot spot

For determining the “final” hot spot we corrected the results of the manually determined hot spots by the calculated CoG by adding both matrix values for x‐ and y‐coordinates per subject and averaging the results.

With respect to the location of the CoG as well as the final hot spot, we performed two‐factorial ANOVAs including the independent factors pulse configuration (monophasic vs. biphasic) and pulse direction (anterior–posterior vs. posterior–anterior) and the dependent factor of x‐coordinate and y‐coordinate, respectively. In doing so, we once took into account the first 37 points stimulated and then all available values in a second calculation. Since three missing values – one for each factor aside of MoP – reduced the power of our analysis of the nine participants, we decided to perform an imputation of these values by the mean of the respective factor. We then performed a second calculation of the final hot spot including these additional values.

#### Intermuscular topography

Testing for differences between the locations of the different muscles, we combined the x‐ and y‐coordinates of each pulse configuration per muscle and calculated repeated measures ANOVA with the dependent variable of the x‐ and y‐coordinate, respectively, and the independent variable type of muscle. Paired two‐sample *t*‐tests corrected for multiple testing were performed for post hoc testing.

Since regularly there were few or no MEPs evoked at the BB‐EMG, this muscle was excluded from topographic statistical analysis.

#### Software

The Software Nucursor (J.C. Rothwell, University College London) was used for the calculation of the MEP amplitude. For statistical analysis, we used IBM's SSPS^®^ version 22 and Microsoft Excel^®^ (Office 2013^®^).

## Results

### Basic measures of excitability

**Figure 3:**
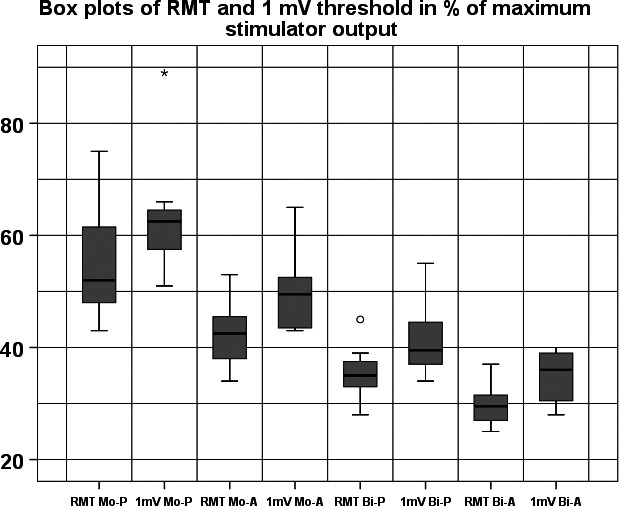
Differences in the stimulation thresholds for the resting motor threshold (RMT) and the 1‐mV threshold. Mo‐P, monophasic posterior current flow; Mo‐A, monophasic anterior current flow; Bi‐P, biphasic pulse with a posteriorly directed second cycle; Bi‐A, biphasic pulse with a anteriorly directed second cycle. The ordinate shows percent of maximal stimulator output. Black asterisks indicate significant difference of the RMT and gray asterisks between the 1‐mV thresholds between any pulse type.

The percentage of maximum stimulator output (MSO) at MEP thresholds and when MEPs were 1 mV amplitude at the hot spot of the FDI were significantly different across current direction (*F*_(1,7)_ = 12.704, *P* = 0.009 and *F*_(1,7)_ = 40.048, *P* < 0.001) and pulse configurations (*F*_(1,7)_ = 150.306, *P* < 0.001 and *F*_(1,7)_ = 132.713, *P* < 0.001), while an interaction between them was found (*F*_(1,7)_ = 39.436, *P* < 0.001 and *F*_(1,7)_ = 43.043, *P* < 0.001). Bi‐A directed current had the lowest (30.36 ± 3.35 and 35.73 ± 4.29% of MSO), Bi‐P directed current had the second lowest (35.44 ± 4.67 and 41.33 ± 6.32% of MSO), Mo‐A current the next lowest (42.7 ± 5.7 and 50.9 ± 6.89% of MSO) and Mo‐P directed current the highest (53.91 ± 9.76 and 64.91 ± 10.34% of MSO) stimulation thresholds of the resting motor potential and the 1‐mV‐threshold potential, respectively. Post hoc t‐tests demonstrated significant differences between all pairs of conditions (*P* = 0.002 for the comparison between Bi‐A and Bi‐P for the MSO when MEPs where 1 mV and *P* < 0.001 for all others) (Figure 3). Due to these substantial differences in 1‐mV stimulation thresholds, we had to manually determine a new hot spot of the 1‐mV potential for each stimulus condition. Mean amplitudes of MEPs were: 1.1 ± 0.3 mV for Mono‐P, 1.1 ± 0.3 mV for Mono‐A, 1.38 ± 0.29 mV for Bi‐A and 1.01 ± 0.29 mV for Bi‐P. When taking into account the first seven stimulations at the hot spot performed for the motor mapping values were as follows: 1.16 ± 0.7 mV for Mono‐P, 1.28 ± 0.63 mV for Mono‐A, 1.06 ± 0.36 mV for Bi‐A and 1.08 ± 0.33 mV for Bi‐P.

### Size of motor representation

There were no significant differences between the four different stimulation modes regarding the overall number of stimulations performed on average (*F*_(1,7_ = 0.053; *P* = 0.825 for pulse configuration, *F*_(1,7)_ = 0.134; *P* = 0.725 for pulse direction) (Table 1). In addition, neither for the first 37 nor all points did pulse configuration (*F*_(1,7)_ = 0.296; *P* = 0.603 and *F*_(1,7)_ = 0.1; *P* = 0.761), pulse direction (*F*_(1,7)_ = 0.035; *P* = 0.858 and *F*_(1,7)_ = 0.414; *P* = 0.540) or an interaction between both (*F*_(1,7)_ = 0.629; *P* = 0.454 and *F*_(1,7)_ = 0.002; *P* = 0.966) alter the sum of mean‐MEPs of any muscle representation. Therefore, we did not find a systematic difference in the size of the representation of the muscles with respect to the stimulus condition.

**Table 1:** Basic statistics of the number of stimulations per stimulation mode

	Sample size	Total number	Minimum	Maximum	Mean ± SD
Mo‐P	11	913	41	166	82.45 ± 33.1
Mo‐A	10	785	61	110	78.5 ± 13.9
Bi‐P	10	827	52	115	82.1 ± 20.76
Bi‐A	10	807	50	120	80.7 ± 21.46

Mo‐P, monophasic posterior current flow; Mo‐A, monophasic anterior current flow; Bi‐P, biphasic pulse with a posteriorly directed second cycle; Bi‐A, biphasic pulse with an anteriorly directed second cycle.

A significant effect regarding the sum of mean‐amplitudes was found for the factor muscle, as expected, with respect to the first 37 points (*F*_(3,21)_ = 21.898; *P* < 0.001) as well as for all points stimulated (*F*_(3,21)_ = 20.176; *P* < 0.001), while an interaction of muscle with pulse configuration (*F*_(3,21)_ = 0.213; *P* = 0.886 and *F*_(3,21)_ = 0.866; *P* = 0.474) or pulse direction (*F*_(3,21)_ = 0.815; *P* = 0.5 and *F*_(3,21)_ = 0.931; *P* = 0.443) was absent. Post hoc testing showed that the sum of means of MEPs differed between all muscle‐representations (*P* < 0.001), except for the comparison of ADM and ECR representation (*P* = 0.70 when considering the central 37 points and *P* = 0.99 when considering all stimulated points).

### Location of the motor representation

**Figure 4:**
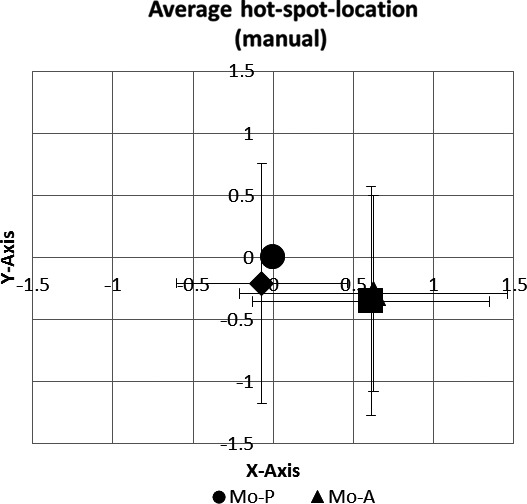
Matrix showing the averaged absolute distances of the manually determined hot spots for each pulse configuration in relation to Mo‐P in cm. No significant differences were found between theses manually determined hot spot locations.

There were no significant differences regarding the locations of the x‐coordinate (*F*_(2,10)_ = 1.108; *P* = 0.368) or the y‐coordinate (*F*_(2,10)_ = 1.116; *P* = 0.355) of the “primary”, i.e., manually determined, hot spots between the different modes of stimulation (Figure 4).

**Figure 5:**
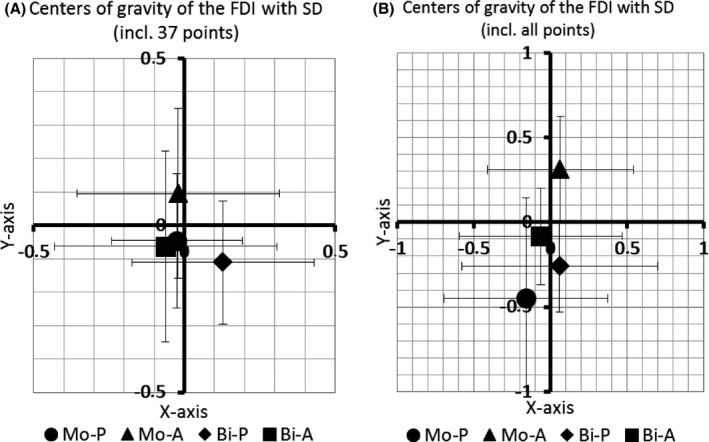
(A) Deviations in cm of the centers of gravity of the first dorsal interosseous muscle from the manually determined hot spot when taking into account the 37 most central matrix points only. Error bars indicate the standard deviation. (B) Deviations in cm of the centers of gravity of the first dorsal interosseous muscle from the manually determined hot spot when taking into account all matrix‐points. Error bars indicate the standard deviation.

Likewise, neither the x‐ nor the y‐coordinate of the CoG taking into account the first 37 matrix points did differ significantly with respect to pulse configuration (*F*_(1,7)_ = 0.121, *P* = 0.738 and *F*_(1,7)_ = 4.276, *P* = 0.077, respectively), pulse direction (*F*_(1,7)_ = 0.054, *P* = 0.823 and *F*_(1,7)_ = 0.707, *P* = 0.428, respectively) and interaction between both (*F*_(1,7)_ = 0.5, *P* = 0.502 and *F*_(1,7)_ = 0.242, *P* = 0.638, respectively) (Figure 5A). For the x‐coordinate we obtained the same outcome when looking at all matrix points with respect to pulse configuration (*F*_(1,7)_ = 0.985, *P* = 0.354), pulse direction (*F*_(1,7)_ = 0.131, *P* = 0.729) and interaction between both (*F*_(1,7)_ = 0.531, *P* = 0.490). However, for the y‐coordinate we found significant differences in the factor pulse configuration (*F*_(1,7)_ = 7.017, *P* = 0.033) and pulse direction (and *F*_(1,7)_ = 13.085, *P* = 0.009) while there was no significant interaction between both (*F*_(1,7)_ = 0.028, *P* = 0.873) (Figure 5B). A post hoc *t*‐test demonstrated a significant difference only for the comparison between MoA and BiP (*P* = 0.002). With respect to the midline, the mean distance of the angle between both parts of the figure‐of‐eight coil and the median‐sagittal line was 2.5–3 cm as determined manually.

**Figure 6:**
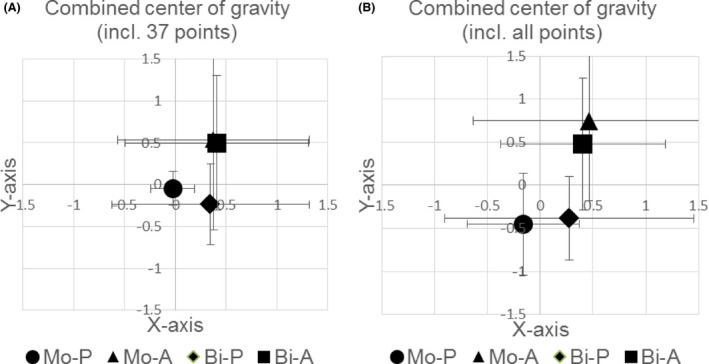
(A) Final center of gravity of the first dorsal interosseous muscle combining the manually determined hot spot as well as the calculated center of gravity, taking into account the 37 first inner matrix points. Error bars indicate the standard deviation. (B) Final center of gravity of the first dorsal interosseous muscle combining the manually determined hot spot as well as the calculated center of gravity, taking into account all measured matrix points. Error bars indicate the standard deviation.

To finally determine the “true hot spot” or center of gravity, we combined the manual measurements of the hot spot as shown in Figure 4 with the correcting calculations of the center of gravity given in Figure 5A and B. We found no significant variation, not when taking into consideration only the inner 37 points for the x‐ and y‐coordinate with respect to pulse configuration (*F*_(1,5)_ = 0.116, *P* = 0.747 and *F*_(1,5)_ = 2.317, *P* = 0.188, respectively), pulse direction (*F*_(1,5)_ = 1.681, *P* = 0.251 and *F*_(1,5)_ = 2.606, *P* = 0.167, respectively) and their interaction (*F*_(1,5)_ = 0, *P* = 0.989 and *F*_(1,5)_ =  0.082, *P* = 0.786, respectively) (Figure 6A), nor when taking into account all available points of stimulation with respect to pulse configuration (*F*_(1,5)_ = 0.555, *P* = 0.490 and *F*_(1,5)_ = 2.673, *P* = 0.163, respectively), pulse direction (*F*_(1,5)_ = 1.232, *P* = 0.317 and *F*_(1,5)_ = 6.390, *P* = 0.053, respectively) and interaction between both (*F*_(1,5)_ = 0.004, *P* = 0.953 and *F*_(1,5)_ = 3.894, *P* = 0.105, respectively) (Figure 6B). However, when compensating for three missing values by means of an imputational method and taking into account all points the effect of the pulse direction on the location of the y‐coordinate was found to be significant (*F*_(1,8)_ = 13.017; *P* = 0.007). Notably, for this calculation the values of the Mo‐P condition were the same as for the CoG calculation, since this condition was used as a reference condition for the manual hot spot determination and hence its coordinates of the manual hot spot were *x* = 0 and *y* = 0.

### Intermuscular topography

**Figure 7:**
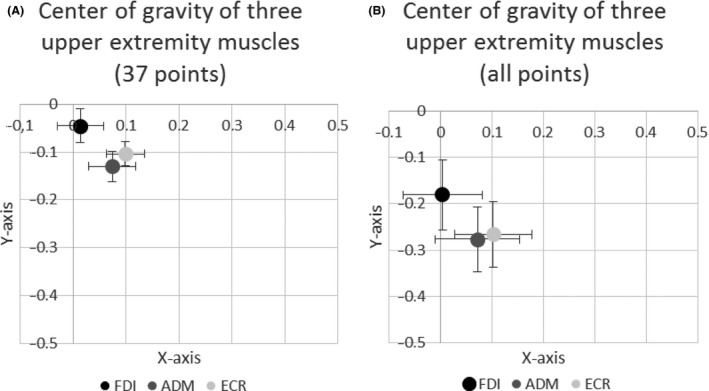
(A) Center of gravity within a Cartesian matrix of the three upper extremity muscles FDI (first dorsal interosseous), ADM (abductor digiti minimi) and ECR (extensor carpi radialis). Bars indicate standard error of the mean. The scale is in cm. (B) Center of gravity within a Cartesian matrix of the three upper extremity muscles FDI (first dorsal interosseous), ADM (abductor digiti minimi) and ECR (extensor carpi radialis). Bars indicate standard error of the mean. The scale is in cm.

In a next step we compared, again separately for the x‐ and y‐coordinate, the effect of the muscles FDI, ADM, ECR on the coordinates. When the coordinates of the three muscles FDI, ADM, and ECR were compared statistically, combining the results from all pulse configurations, we found highly significant differences in x‐coordinate (*F*_(2,39)_ = 8.756; *P* < 0.001 for the first 37 points and *F*_(2,39)_ = 7.606; *P* = 0.001 for all points) as well as for y‐coordinates (*F*_(2,39)_ = 11.541; *P* < 0.001 for the first 37 points and *F*_(2,39)_ = 11.909; *P* < 0.001) (Figure 7A and B). In this case, post hoc testing revealed significant differences between the FDI and the ADM as well as ECR coordinates with respect to x‐ and y‐coordinates, regardless of the total number of measured points that were taken into account. No significant difference was found between the coordinates of ADM and ECR.

## Discussion

### Effect of the pulse modality

In this study we investigated the effect of the pulse‐configuration and direction of single‐pulse TMS on the location of the center of gravity of muscles of the hand and the arm. Taking into account monophasic and biphasic pulses with an anterior‐posteriorly or posterior‐anteriorly oriented pulse configuration, respectively, we did not find any significant differences between these four pulse configurations regarding the overall size of the muscle representations, but found that the cranio‐caudal location of the final hot spot did depend on the pulse direction when all stimulated points were included into the calculation. Hence, current direction is relevant for motor mapping with TMS. Consequently, anteriorly directed pulses tend to localize more anterior as compared to posteriorly directed pulses.

The different properties of the pulse modalities have been linked to different intracortical patterns of excitation. Based on studies with epidural spinal recordings demonstrating different latencies of the induced potentials, monophasic pulses with anterior current direction would induce an indirect wave (I_1_‐wave) which in the case of high stimulus intensities is complemented by additional I‐waves that will finally induce a direct pyramidal cell activation (D‐wave) (Di Lazzaro et al. 1998; Salvador et al. 2011). In the case of a monophasic current of posterior current direction, later I‐waves and D‐waves will be recruited (Di Lazzaro et al. 2001). The pattern of activation of a biphasic pulse with a first phase of anterior and a second phase of posterior direction is similar to that. Likewise, the recruitment pattern of biphasic pulses with a first phase of posterior and a second phase of anterior direction is similar to that of monophasic pulses of anterior current direction (Di Lazzaro et al. 2001). Our results indicate that the net effect in the form of excitation of corticomotoneuronal cells by TMS is not significantly altered by the pulse configuration but its orientation. They therefore confirm that the pulse direction influences the way how descending action potentials are induced (Thompson et al. 1990). However, the relatively discrete changes in the location of the hot spot indicate that TMS may still act on a mechanistically robust representation of muscles or movements in the primary motor cortex. Still, if there is something like a robust cortical representation of a muscle the question arises which pulse direction induces the more accurate estimate of the motor cortex representation. The posteriorly directed pulses induce a current flow from cortical layer VI to I and may be able to excite axons directly and most likely closely to the place of their generation. In contrast, anteriorly directed pulses propagate from layer I to VI and may therefore induce a greater spread of activation, e.g., via excitation of fibers of the molecular layer (Jefferys 1981).

In line with available evidence, the stimulation thresholds of the four different stimulus configurations in this study differed significantly, with the biphasic stimulation modes having lower thresholds than the thresholds measured with monophasic pulses (Sommer et al. 2006). The overall lowest thresholds were found with the biphasic anterior–posteriorly oriented pulses and the highest thresholds with the monophasic anterior–posteriorly oriented pulses. This also accords with the available literature, which generally reports the high effectiveness of those currents having a primarily posterior to anterior or lateral flow in the brain, even though the data indicate inter‐individual differences regarding the most effective type of stimulation (Balslev et al. 2007). The overall greater effectiveness of biphasic stimuli is due to the frequently reported higher efficiency of the second phase of the biphasic pulse as compared to its first phase or to monophasic pulses (Brasil‐Neto et al. 1992; Salvador et al. 2011). Since sensitivity to the direction of the current has been found to be highest in monophasic pulse configurations, such pulses have been thought preferable in studies for mapping of the motor cortex (Brasil‐Neto et al. 1992).

In general, a manual determination of the hot spot appears to provide a good approximation of the “true hot spot” as determined by calculation of the center of gravity. All of our CoGs were found within a radius of one cm around the manually determined hot spot, which supports results by Wassermann et al. (1996).

### Intermuscular topography

Regarding the topographic distribution of FDI, ADM, and ECR, our results are well in line with the common somatotopic motor map of a left hemisphere which shows the first dorsal interosseous muscle, i.e., a muscle concerned with abduction of the index finger, as being located most laterally – whereas the abductor digiti minimi, i.e., a fifth‐finger muscle, as well as the extensor carpi radialis, i.e., a hand/wrist muscle, were found to be located more medially. The fact that we did not find significant differences between the location of ADM and ECR, representing respectively a fifth‐finger muscle and a hand muscle, is most likely due to their topographic proximity in a cortical representation in which, medially to laterally, the forearm muscles are followed by the hand muscles and then by those of the fingers V to I (Penfield and Rasmussen 1950). Hence, the inter‐individual somatotopic resolution of TMS as performed in our study may not be sufficient to detect the smallest spatial borders like those between the fifth finger and hand muscle representations but was able to detect the difference between somatotopically more distant representations such as those of the index finger and the hand. In total numbers the distance between the centers of gravity was found to be approximately only 1 mm, which again may reflect the high variability between the representations (Melgari et al. 2008). As well, it had been demonstrated that the representation of finger muscles can largely overlap (Indovina and Sanes 2001). Therefore, it may be particularly difficult to detect differences in the recruitment pattern using TMS, this being a method likely to result in wide activation of brain tissue. However, our data also indicate that differentiating between the categories “hand/finger” and “forearm‐muscles” may not be sufficient when analyzing somatotopy (Melgari et al. 2008). In fact, the cortical representation of single finger muscles may occupy larger cortical areas than other much larger body parts even of the upper extremity. Hence, high degrees of cortical overlapping and co‐activation between hand and forearm muscles may well explain the spatially only small differences that we found in this study, but do not take into consideration the significant difference between the index finger and the small finger muscle representation that can be found. Interestingly, we not only found significant differences in the representation of the FDI with respect to ADM and ECR muscle distribution in the medio‐lateral direction represented by the *x*‐axis of our coordinate system, but also with regard to the anterior–posterior location represented by the *y*‐axis. Earlier work suggests that the representation of extensor muscles is generally more anterior as compared to flexor muscles (Foerster 1936). Although there are several studies of high‐resolution electrocortical stimulation in non‐human primates, there is little evidence from studies in humans due to limitations in the spatial resolution of the commonly used electrodes. In the classic view, the primary motor cortex, which is also designated as M1, is defined histologically by a preponderance of large pyramidal neurons in cortical layer III and more so in layer V and less developed granular layers, giving this structure a distinct appearance. This type of cortex is mainly located in the anterior wall of the precentral gyrus and only in more dorsal parts also on its crown (Geyer et al. 1996). Hence, a more superficial location may correlate with the overall larger representation of the FDI compared to the ADM and ECR. However, no formal mapping for the ECR and ADM was done, and hence the CoG was biased toward the FDI muscle a priori. In addition, unspecific activation of other thenar muscles may have facilitated this finding.

In a recent work, modeling the effects of transcranial magnetic stimulation on distinct neuronal elements, four main sites of action were determined, namely (1) terminations of medium‐caliber horizontal fibers within the crown of a gyrus and parallel to the induced electric field, (2) terminations of medium‐caliber intracortical axons, vertical pyramidal axon collaterals and axon terminations in the lip of the gyrus and nearby deep in the sulcus, (3) at bends of pyramidal fibers with larger diameters in the white matter below the lip of the gyrus, and 4) in Betz cells along the surface of the vertical wall of the sulcus at a depth of at least 1.5 cm below the cortex (Silva et al. 2008; Salvador et al. 2011). Moreover, the tissue heterogeneity was found to be a major factor in the accuracy of predictions. This demonstrates that the actual effect of the TMS depends on the complex composition of the cortical compounds within the electrical field. Still, proximity to the cortical surface and to a gyral lip may facilitate efficacy of TMS regardless of the configuration and direction of the pulses.

### Limitations

Limitations of this study include the fact that stimulation was performed purely manually. As it has been shown that MRI‐guided neuronavigation can improve the reproducibility and accuracy of motor maps and we may assume that using such a technique could have influenced the results of this study, possibly by assisting in discerning the predetermined hot spots for the different stimuli (Herwig et al. 2002; Bashir et al. 2013). Because of the high number of stimulated matrix points and the nature of the calculation of the center of gravity, we consider our data still representative. Regarding the accuracy of our measurements, we have to take into account that the maximum number of stimulations per point was limited to 7. This is a relatively low number of repeats and below the optimal number of repetitions for finding representative MEPs, this having been determined to be 20 (Classen et al. 1998). However, the same study also demonstrated the high accuracy of a lower number of stimulations in relation to the number of total stimulation points. In addition, in the calculation of the final hot spot we decided to adjust for missing values using a method of imputation. By that, even though we increased the power of our study we also increased the likelihood of an alpha error.

Assuming a high degree of variability and therefore rather small differences between alternating pulse configurations a higher number of participants may be required to test for this hypothesis. Generally, our diagram as well as what is currently understood regarding the mode of action of TMS pulses would suggest that in such a map Mo‐P and Bi‐P pulse configurations may result in relatively similar representations as well as Mo‐A and Bi‐A pulse configurations. In conclusion, the pattern of the cortical representation of the first dorsal interosseous muscle is independent of TMS pulse configuration. Hence, the choice of the stimulus modalities may have a limited influence on the validity of TMS motor mapping. Motor mapping with TMS demonstrates agreement here with classic somatotopic distribution, while spatial resolution of the technique may be restricted.

## Conflict of Interest

The authors have nothing to disclose.
